# Elevated Serum Lactate in Glioma Patients: Associated Factors

**DOI:** 10.3389/fonc.2022.831079

**Published:** 2022-05-19

**Authors:** Beathe Sitter, Annamaria Forsmark, Ole Solheim

**Affiliations:** ^1^ Department of Circulation and Medical Imaging, NTNU, Norwegian University of Science and Technology, Trondheim, Norway; ^2^ Department of Anaesthesia and Intensive Care, Nord-Trondelag Health Trust, Levanger, Norway; ^3^ Department of Neurosurgery, St. Olav’s Hospital, Trondheim University Hospital, Trondheim, Norway; ^4^ Department of Neuromedicine and Movement Science, Faculty of Medicine, NTNU, Norwegian University of Science and Technology, Trondheim, Norway

**Keywords:** glioma, lactate, hyperlactatemia, biomarker, corticosteroid

## Abstract

**Introduction:**

Serum lactate levels in brain cancer patients correlate with tumor malignancy grading, and serum lactate has been suggested as a potential biomarker and prognostic factor. The purpose of this study was to identify potential sources of elevated serum lactate in patients with brain gliomas by examining factors of importance for serum lactate production and clearance.

**Methods:**

In this cross-sectional study, data were collected from 261 glioma patients who underwent surgery from March 2011 to June 2015. We recorded patient gender, age, blood serum measures of lactate, glucose, pH, hemoglobin and base excess, patient health status, medications, and tumor characteristics. Patients with elevated and normal serum lactate levels were compared, and we explored if there were correlations between the variables. The association of serum lactate with the measured variables was investigated by simple and multivariable linear regression models.

**Results and Discussion:**

Patients with elevated serum lactate had higher blood glucose, larger tumor volumes, and more tumor edema; more often needed pressor medication during surgery; and more often received corticosteroid treatment. The investigated variables were highly correlated. Multivariable linear regression indicated that gender, tumor volume, Charlson Comorbidity Index, hyperglycemia, and corticosteroid treatment were associated with serum lactate levels. Histopathology was not an independent factor. In conclusion, comorbidities, hyperglycemia, and presurgical corticosteroid treatment exhibited the strongest association with serum lactate in glioma patients.

## 1 Introduction

Glioma is the most common primary brain tumor (1). Gliomas are classified into four groups based on their histopathological and molecular features (World Health Organization classification of tumors of the central nervous system; WHO 1-4) (2). This grading predicts clinical behavior and is key for treatment decisions. The most malignant form (WHO 4) is glioblastoma multiforme, which has a dismal prognosis (3). Only 3%–5% of glioblastoma patients survive 5 years or more, and this low survival rate has changed little in the past decades (4).

Blood serum lactate has been proposed as a biomarker for tumor malignancy and a prognostic factor for brain tumor patients (5–11). Hyperlactatemia (blood serum lactate >2 mmol/L) has been reported more frequently in patients with high-grade than low-grade brain tumors (5, 6, 8, 11). Also, higher blood serum levels have been found in high-grade glioma (HGG) compared to low-grade glioma (LGG) patients, and patients with elevated blood serum lactate (≥2 mmol/L) had shorter progression-free survival (9). It has been suggested that the production of lactate in the tumor could be the source of elevated blood serum lactate in patients with malignant brain tumors (5, 8). It is well documented that malignant brain tumors can produce lactate not only by anaerobe glucose metabolism but also by conversion of glucose to lactate in the presence of oxygen, known as the Warburg effect (12–14). However, it is not known to what extent this lactate production is reflected in the serum.

Several patient-, tumor-, and treatment-related factors can increase serum lactate levels by increasing production and/or reducing clearance (15). Other conditions and their treatments, like diabetes mellitus, can also impact serum lactate levels. The aim of this study was to examine potential patient-, tumor-, and treatment-related factors for their association with elevated serum lactate in patients with glioma and to explore potential causes of elevated serum lactate in these patients.

## 2 Materials and Methods

### 2.1 Study Population

In this cross-sectional study, we collected data from patient files and anesthesia records from a local registry of patients who underwent surgical biopsy or resection for intracranial tumors. We screened patients treated for brain tumors from March 2011 to June 2015 at St. Olav’s Hospital, Trondheim University Hospital, and we identified patients with documented serum lactate measures in their anesthesia journal (N = 659). Patients with other tumor entities than diffuse glioma WHO grades 2–4 (N = 392) and patients in whom blood gas analysis was done more than 30 min after surgery started (N = 6) were excluded. A total of 261 patients with diffuse gliomas verified by histopathology were included in the study. The median age of the patients was 59 years, ranging from 18 to 84 years. Demographic data of the patient cohort are presented in **Table 1**. The study was approved by the Regional Committees for Medical and Health Research Ethics (REC-Id 2015/1181). All patients have signed informed consent for the data registration.

**Table 1:** Age and gender of patients diagnosed with low-grade (WHO 1 and 2) and high-grade (WHO 3 and 4) glioma at time of surgery (N = 266).

	*Low-grade glioma*	*High-grade glioma*	
	WHO 2	WHO 3	WHO 4
*Median age (range) in years*	44 (18–78)	56 (34–82)	62 (19–84)
*Women, N (%)*	27 (26.7%)	14 (13.9%)	57 (56.4%)
*Men, N (%)*	39 (23.6%)	20 (12.1%)	104 (63.0%)
*Total*	66	34	161

### 2.2 Data Collection

We recorded serum lactate and measures of patient-related, tumor-related, and treatment-related factors. This included patient gender and age, analysis of blood gasses (serum lactate, glucose, hemoglobin (Hb), base excess (BE), and pH), presurgical volumes of tumors, the largest diameter of tumor edema calculated from MR images, tumor histopathology according to the 2007 and 2016 WHO classification (2, 16), if it was a primary operation for a brain tumor, and preoperative Karnofsky performance scale (17) and Charlson Comorbidity Index (CCI) (18) if a patient had hyperglycemia, diabetes, intraoperative hypotension, pressor treatment during tumor surgery, and presurgical treatment with corticosteroids. The Karnofsky performance scale, ranging from 100 to 0, classifies patients according to their functional impairment, with high scores for high functionality (17). CCI is based on 17 categories of medical conditions (18). Higher scores predict a higher risk for mortality or resource use. CCI values were dichotomized between 0–1 and 2 or higher. Tumor histopathology was dichotomized to LGG for WHO grade 2 and to HGG for WHO grades 3 and 4. The blood samples were collected within the first 30 min of surgery, after induction of anesthesia but before removal of the tumor. The samples were analyzed within 10 min using the ABL800 FLEX (Radiometer Medical ApS, København, Denmark).

### 2.3 Statistical Analysis

#### 2.3.1 Univariate Analysis for Serum Lactate

Patient age and gender, blood gasses (glucose, hemoglobin (Hb), BE, and pH), tumor characteristics (tumor volume, tumor edema, and dichotomized histopathological grading), whether it was primary surgery, other conditions and diseases (Karnofsky performance scale, dichotomized CCI, hyperglycemia, diabetes, and intraoperative hypotension), and medication (intraoperative pressor and corticosteroids) were compared for patients with normal (≤1.6 mmol/L) and with elevated serum lactate (>1.6 mmol/L). Continuous variables were checked for normality by QQ plots. Age, Hb, BE, pH, and Karnofsky performance scale demonstrated the normal distribution of values, whereas glucose, tumor volume, and tumor edema all demonstrated distribution that deviated from normal. We thus used Student’s t-test and median test to compare mean values for continuous variables between groups of patients accordingly and by Fisher’s exact test for all dichotomous variables. Statistical significance was set to p < 0.05, and corrections for multiple hypothesis testing were done with the Bonferroni method.

#### 2.3.2 Correlation Analysis

Correlation between variables was investigated by Pearson’s correlation analysis when including continuous variables and by Fisher’s exact test and Spearman’s rho for dichotomous variables.

#### 2.3.3 Linear Regression

Simple linear regression models for serum lactate were calculated for all variables that could predict serum lactate elevation. These were the patient’s gender, age, tumor volume, tumor edema, dichotomized tumor grading, whether it was a primary operation, Karnofsky performance scale, dichotomized CCI, hyperglycemia, diabetes, intraoperative hypotension, and treatment with pressor during anesthesia/tumor surgery or presurgical corticosteroids.

All variables that had coefficients significant at a 0.20 level in the simple regression models were included in the multivariable linear regression model. The multivariable linear regression model was stepwise adjusted by removing variables with the largest p-value until all variable coefficients in the model were significant at a 0.10 level.

All statistical analyses were conducted in Statistical Package for Social Sciences (IBM SPSS Statistics, version 28).

## 3 Results

### 3.1 Univariate Analysis for Serum Lactate

Mean values with SD for continuous variables and frequencies for dichotomous variables are presented in **Table 2** for patients with normal (≤1.6 mmol/L) and elevated (>1.6 mmol/L) serum lactate. **Table 2** shows statistically significant differences after Bonferroni correction (q < 0.05) for seven of the measured variables. Patients with elevated serum lactate had higher blood glucose and larger tumor volume and tumor edema and were more frequently diagnosed with hyperglycemia and diabetes and treated with pressor and corticosteroids as compared to patients with normal serum lactate levels.

**Table 2:** Variable values for patients with normal (≤1.6 mmol/L) and elevated (>1.6 mmol/L) values of serum lactate, given as mean values with SD for continuous variables, and as frequency of patients in numbers and percentages for dichotomous variables.

*Factor*	*N*	*Serum lactate ≤1.6 mmol/L*		*Serum lactate >1.6 mmol/L*		*p*	*q*
*Gender, male (N, %)*	261	105	59%	58	70%	0.101	1
*Mean age (years)*	261	56 ( ± 15)		59 ( ± 13)		0.120	1
*Glucose (mmol/L)*	261	6.3 ( ± 1.5)		7.6 ( ± 2.1)		**<0.001**	**0.006**
*Hemoglobin (mmol/L)*	261	12.9 ( ± 1.3)		13.2 ( ± 1.1)		**0.015**	0.258
*Base excess (mmol/L)*	261	0.14 ( ± 1.88)		0.84 ( ± 2.04)		**0.007**	0.113
*pH*	261	7.42 ( ± 0.05)		7.42 ( ± 0.05)		0.869	1
*Tumor volume (cm^3^)*	240	25.4 ( ± 30.7)		39.6 ( ± 37.1)		**0.001**	**0.016**
*Edema max diameter (mm)*	229	9.2 ( ± 11.8)		15.4 ( ± 14.2)		**<0.001**	**0.011**
*High-grade glioma (N, %)*	261	124	70%	69	83%	**0.023**	0.394
*Primary operation (N, %)*	252	100	58%	53	67%	0.168	1
*Karnofsky performance scale*	171	84 ( ± 15)		77 ( ± 15)		**0.012**	0.206
CCI *> 1 (N, %)	240	7	4%	7	9%	0.136	1
*Hyperglycemia (N, %)*	233	7	4%	14	20%	**<0.001**	**0.003**
*Diabetes (N, %)*	240	4	2%	9	12%	**0.002**	**0.035**
*Hypotension (N, %)*	261	20	11%	6	7%	0.380	1
*Pressor (N, %)*	261	131	74%	44	53%	**<0.001**	**0.017**
*Corticosteroids (N, %)*	240	62	37%	58	78%	**<0.001**	**<0.001**

Differences for patients with normal and elevated serum lactate were evaluated by Student’s t-test or median test for normal and non-normally distributed continuous variables, respectively, and by Fisher’s exact test for dichotomous variables. Measures of statistical significance are given by p-value, and by q-value correcting for multiple hypothesis testing. Statistically significant p- and q-values are written in bold types.

^*^CCI, Charlson Comorbidity Index.

### 3.2 Correlation Analysis

Correlation coefficients of all investigated variables are shown with a heatmap in **Table 3**. All variables correlated with at least one other variable. pH had the lowest number of correlations, correlating only with BE. Nine of the variables (serum lactate, age, blood glucose, BE, tumor volume, tumor edema, glioma grading, Karnofsky performance scale, and treatment with corticosteroids) correlated with half or more of the other variables. Most correlations were weak (0.10–0.39), and the strongest correlations were moderate (0.40–0.69). Serum lactate had one moderate correlation (0.477), to corticosteroid treatment.

**Table 3:** Correlation matrix with heatmap of coefficients between all variables.

	Lac	Gndr	Age	Glc	Hb	BE	pH	TVol	Oedm	HGG	PrimO	Krnfsk	CCI	HprGlc	Diab	Hypot	Press	Strds
**Lac**	1	0.100	**0.141**	**0.384**	**0.189**	**0.221**	−0.005	**0.261**	**0.258**	**0.226**	0.109	**−0.268**	**0.145**	**0.281**	**0.173**	−0.085	**−0.198**	**0.475**
**Gndr**	0.100	1	−0.013	0.021	**0.418**	−0.010	−0.031	0.056	−0.016	0.044	0.058	0.109	−0.034	0.109	0.104	−0.033	−0.055	−0.009
**Age**	**0.141**	−0.013	1	**0.229**	−0.040	**0.161**	0.006	0.065	**0.333**	**0.421**	**0.246**	**−0.392**	**0.137**	**0.131**	0.031	**−0.128**	**0.158**	**0.320**
**Glc**	**0.384**	0.021	**0.229**	1	0.099	0.039	−0.108	**0.148**	**0.213**	**0.243**	0.051	**−0.293**	**0.164**	**0.679**	**0.649**	−0.057	−0.083	**0.384**
**Hb**	**0.189**	**0.418**	−0.040	0.099	1	**0.132**	−0.113	0.101	0.023	0.102	**0.205**	−0.077	−0.082	0.046	−0.002	−0.034	−0.060	**0.169**
**BE**	**0.221**	−0.010	**0.161**	0.039	**0.132**	1	**0.413**	**0.226**	**0.249**	**0.182**	0.105	**−0.238**	0.005	0.102	0.054	−0.100	**−0.126**	**0.309**
**pH**	−0.005	−0.031	0.006	−0.108	−0.113	**0.413**	1	−0.010	0.072	0.016	−0.020	−0.042	−0.074	−0.047	−0.017	−0.016	0.011	0.015
**TVol**	**0.261**	0.056	0.065	**0.148**	0.101	**0.226**	−0.010	1	0.119	0.085	**0.189**	**−0.388**	0.008	0.073	**0.147**	−0.104	**−0.133**	**0.261**
**Oedm**	**0.258**	−0.016	**0.333**	**0.213**	0.023	**0.249**	0.072	0.119	1	**0.451**	0.013	**−0.445**	0.021	**0.173**	0.00	**−0.164**	**−0.217**	**0.491**
**HGG**	**0.226**	0.044	**0.421**	**0.243**	0.102	**0.182**	0.016	0.085	**0.451**	1	**0.131**	**−0.478**	0.034	**0.131**	0.024	**−0.152**	−0.100	**0.504**
**PrimO**	0.109	0.058	**0.246**	0.051	**0.205**	0.105	−0.020	**0.189**	−0.013	**0.131**	1	0.048	0.060	−0.041	−0.028	−0.005	−0.017	**0.263**
**Krnfsk**	**−0.268**	0.109	**−0.392**	**−0.293**	−0.077	**−0.238**	−0.042	**−0.388**	**−0.445**	**−0.478**	0.048	1	−0.031	**−0.171**	−0.138	0.149	0.088	**−0.374**
**CCI**	**0.145**	−0.034	**0.138**	**0.164**	−0.082	0.005	−0.074	0.008	0.021	0.034	0.060	−0.031	1	0.110	**0.255**	0.090	0.023	0.071
**HprGlc**	**0.281**	0.109	**0.131**	**0.679**	0.046	0.102	−0.047	0.073	**0.173**	**0.131**	−0.041	**−0.171**	0.110	1	**0.576**	−0.109	−0.129	**0.226**
**Diab**	**0.173**	0.104	0.031	**0.649**	−0.002	0.054	−0.017	**0.147**	0.00	0.024	−0.028	−0.138	**0.255**	**0.576**	1	−0.021	−0.107	0.055
**Hypot**	−0.085	−0.033	**−0.128**	−0.057	−0.034	−0.100	−0.016	−0.104	**−0.164**	**−0.152**	−0.005	0.149	0.090	−0.109	−0.021	1	**0.233**	**−0.177**
**Press**	**−0.198**	−0.055	**0.158**	−0.083	−0.060	**−0.126**	0.011	**−0.133**	**−0.217**	−0.100	−0.017	0.088	0.023	−0.129	−0.107	**0.233**	1	**−0.239**
**Strds**	**0.475**	−0.009	**0.329**	**0.384**	**0.169**	**0.309**	0.015	**0.261**	**0.491**	**0.504**	**0.263**	**−0.374**	0.071	**0.226**	0.055	**−0.177**	**−0.239**	1

Coefficients were determined by Pearson’s correlation analysis for continuous variables, and Spearman’s rho with Fisher’s exact test between dichotomous variables. Significant coefficients (p < 0.05) are reported in bold.

Lac, serum lactate; Gndr, gender; Glc, glucose; Hb, hemoglobin; BE, base excess; TVol, tumor volume; Oedm, edema maximum diameter; HGG, high-grade glioma; PrimO, primary operation; Krnfsk, Karnofsky performance scale; CCI, Charlson Comorbidity Index; HprGl, Glucose over 10 mmol/L; Diab, diabetes; Hypt, hypotension; Press, pressor; Strds, corticosteroids. The overlayed heatmap indicate strength of correlations, with blue for positive and red for negative correlations. Weak correlations (0.10 – 0.39) are in the lightest shade, moderate correlations (0.40 – 0.69) in medium shade, and very strong correlation (1) in dark shade.

### 3.3 Linear Regression

**Table 4** shows coefficients and p-values from simple linear regression models and the adjusted multivariable linear regression model of serum lactate. With the exception of gender, primary operation, and hypotension, all variable coefficients were significant (p < 0.05) in simple linear predictions of serum lactate. Five of the variable coefficients—gender, tumor volume, dichotomized CCI, hyperglycemia, and corticosteroid treatment— were significant in the adjusted multivariable model at a 0.10 level of significance. The adjusted multivariable regression model described 30.1% of the variation in serum lactate (ANOVA: p < 0.05).

**Table 4:** Variables and their coefficients and p-values for simple and adjusted multivariable linear regression models of serum lactate.

*Variable*	*Simple*		*Multivariable*	
	Coefficient	p	Coefficient	p
*Constant*		0.926	<0.001	
*Gender, male*	0.134	0.109	0.146	0.060
*Age (years)*	0.006	0.023		
*Tumor volume (cm^3^)*	0.005	<0.001	0.003	0.008
*Edema max diameter (mm)*	0.013	<0.001		
*High-grade glioma*	0.336	<0.001		
*Primary operation*	0.145	0.085		
*Karnofsky performance scale*	−0.012	<0.001		
CCI* > 1	0.404	0.024	0.282	0.067
*Hyperglycemia*	0.633	<0.001	0.349	0.008
*Diabetes*	0.499	0.007		
*Hypotension*	−0.186	0.170		
*Pressor*	−0.275	0.001		
*Corticosteroids*	0.619	<0.001	0.505	<0.001

The multivariable model explained 30.1% of the variation in serum lactate. *CCI, Charlson Comorbidity Index.

## 4 Discussion

Patients with elevated serum lactate had higher blood glucose and larger tumor volume and tumor edema and were more frequently diagnosed with hyperglycemia and diabetes and treated with pressor intraoperatively and corticosteroids before surgery than patients with normal levels of serum lactate. Elevation of lactate is seemingly more associated with factors associated with corticosteroid use than histopathology. Lactate does not seem to be an attractive biomarker in glioma.

The adjusted multivariable regression model indicated that male patients with glioma had 0.1 mmol/L higher levels of serum lactate than women. Lactate reference values for men and women are the same (19), and this calculated association between gender and serum lactate levels may be an indirect effect. Tumor volume was found to be statistically significant for serum lactate levels. However, differences in tumor volume were associated with minor changes in serum lactate values (**Table 4**). Patients with CCI of 2 or higher and hyperglycemia would have markedly higher serum lactate levels than patients with CCI scores of 1 or 0 and normal glucose levels. The variable with the largest impact was corticosteroid treatment, as patients on corticosteroid treatment had 0.5 mmol/L higher estimated levels of serum lactate than patients that were not treated with corticosteroids.

The variables investigated for associations with serum lactate levels showed several covariations. This lack of independence of variables in the multiple linear regression can affect the resulting coefficients and p-values. Of the five variables included in the adjusted multivariable model (**Table 4**), gender and CCI were not correlated to other variables in the model, whereas corticosteroid treatment was correlated to tumor volume and hyperglycemia (**Table 3**). The p-values of coefficients for these three variables were highly significant (p ≤ 0.008), and thus we presume the regression model to be valid. Tumor volume, hyperglycemia, and corticosteroid treatment were also found to be significantly different in patients with elevated compared to normal serum lactate (**Table 2**), supporting that these three factors may explain serum lactate levels. Still, there is a risk of overfitting regression models by selecting variables based on p-values. As a scatter plot (not shown) of standardized residuals against predicted values for lactate showed no specific pattern, the model is presumed to not be overfitted.

We observed that elevated serum lactate levels were more frequent in patients with HGG than LGG (**Table 2**), which agrees with the previously reported findings (5, 6, 8, 11). Previously, logistic regression by blood metabolites, adjusted for age, gender, steroid use, and other factors, indicated significantly higher levels of lactate and other metabolites in the blood of patients with HGG (10). We also observed a significant correlation between tumor grading and serum lactate (**Table 3**) and a highly significant single linear regression model for serum lactate with tumor grading as a variable (**Table 4**). However, tumor grading was not significant in the multivariable regression model. Neither were the patient’s age, tumor edema, primary operation, Karnofsky performance scale, diabetes, hypotension, or intraoperative treatment with pressor. Gender, tumor volume, CCI, hyperglycemia, and corticosteroid treatment appear as the most important of the investigated factors for serum lactate levels in glioma patients. Since tumor volume is correlated to gender (20) and corticosteroid treatment, and hyperglycemia may result from corticosteroid treatment, corticosteroid treatment or stress responses may emerge as a common trait.

Tumors can produce lactate also under sufficient oxygen supply (21). Excessive production of lactate is associated with increased aggressiveness, as it acidifies the extracellular environment that induces invasion and metastasis and inhibits the antitumor immune response and resistance to therapy (22). Previous studies with the correlation between tumor malignancy and serum lactate levels have identified serum lactate as a potential biomarker of malignancy of adult brain tumors (5, 8). Our study indicates that although serum lactate is associated with tumor volume and histopathological grading, comorbidities, hyperglycemia, and treatment with corticosteroids have a larger impact. Corticosteroid treatment can release glucose, increase insulin resistance, and increase lactate production (23). Increased serum lactate levels after corticosteroid treatment during cardiac surgery have been reported. Dexamethasone is commonly used in the management of glioma patients to treat peritumoral edema and to control neurological symptoms (24).

The investigated associations of serum lactate levels and patient-, tumor-, and treatment-related factors revealed comorbidity, hyperglycemia, and corticosteroid treatment as the most important factors. These factors are not directly related to tumor biology. Corticosteroid treatment may be a common trait, and it seems unlikely that serum lactate is a relevant biomarker in glioma. The potential for serum lactate as a biomarker in glioma therefore seems low.

There are limitations to this retrospective study. Ideally, serum lactate should have been measured repeatedly over time, prior to surgery and cancer treatments. Also, knowledge about the type of pressor given to patients would have enabled a more precise evaluation of the association between pressor treatment and serum lactate levels, as only specific pressor types affect serum lactate. However, a strength of this study is the exploration and evaluation of relevant patient-, tumor-, and treatment-related factors for their association with serum lactate in a significant number of glioma patients. The presented findings should be important for prospective studies of mechanisms behind elevated serum lactate in brain glioma patients.

In conclusion, hyperglycemia, comorbidities, and presurgical corticosteroid treatment appear as the factors with the strongest association with serum lactate in glioma patients.

## Data Availability Statement

The data analyzed in this study is subject to the following licenses/restrictions: the ethical approval given to the patient registry does not include the transfer of data. Requests to access these datasets should be directed to beathe.sitter@ntnu.no.

## Ethics Statement

The studies involving human participants were reviewed and approved by Regional Committees for Medical and Health Research Ethics. The patients/participants provided their written informed consent to participate in this study.

## Author Contributions

BS, AF, and OS contributed to the conception and design of the study. AF organized the database. BS performed the statistical analysis and wrote the manuscript draft. All authors contributed to manuscript revision and read and approved the submitted version.

## Conflict of Interest

The authors declare that the research was conducted in the absence of any commercial or financial relationships that could be construed as a potential conflict of interest.

## Publisher’s Note

All claims expressed in this article are solely those of the authors and do not necessarily represent those of their affiliated organizations, or those of the publisher, the editors and the reviewers. Any product that may be evaluated in this article, or claim that may be made by its manufacturer, is not guaranteed or endorsed by the publisher.
